# Modern health-seeking behaviour and associated factors among mothers having under 5-years old children in Shire town, Tigray, Ethiopia: A cross-sectional study 2018

**DOI:** 10.4102/phcfm.v11i1.1913

**Published:** 2019-08-21

**Authors:** Girmay T. Weldesamuel, Tsega T. Alemayoh, Hagos T. Atalay, Teklewoini M. Zemichael

**Affiliations:** 1 School of Nursing, Aksum University, Aksum, Tigray, Ethiopia; 2 Department of Medical Laboratory, Aksum University, Aksum, Tigray, Ethiopia

**Keywords:** modern, health care-seeking behaviour, under 5-years old children, mother

## Abstract

**Background:**

The practice of appropriate health care-seeking is important to reduce severe and life-threatening childhood illnesses. In Shire town, little is known about the mother’s health care-seeking behaviour on childhood illness.

**Aim:**

To assess modern health-seeking behaviour and associated factors of mothers having under 5-years old children in Shire town.

**Setting:**

The study setting was Shire town, northwest Tigray, Ethiopia.

**Methods:**

A community-based cross-sectional study was conducted to interview 504 mother-child pairs by systematic random sampling technique. Data were collected through interviewer-administered semi-structured questionnaires. Data were coded, entered, cleaned and edited using EPIDATA version 3.1 and export to Statistical Package for Social Science (SPSS) Version 22.0 for analysis. To identify the significant variables, binary logistic regression was employed. Variables with *p*-value < 0.05 at 95% CI (confidence interval) in multivariate logistic regression were considered statistically significant.

**Results:**

In this study, around 76.2% (72.1, 80) of mothers sought modern health care. In a multivariate logistic regression analysis at a *p*-value of < 0.05, caregivers with age of ≥ 28 years (AOR [adjusted odds ratios]: 1.65; 95% CI [1.02, 2.68]), educational level of secondary school and above (AOR: 0.44; 95% CI [0.23, 0.86]), child feeding per day < 8 times (AOR: 2.77; 95% CI [1.75, 4.38]) and perceived severity of illness (AOR: 2; 95% CI [1.07, 3.82]) were statistically associated with modern health care-seeking behaviour.

**Conclusion:**

Strengthen healthcare services is recommended at the community level through information, education and communication/behavioural change strategies to improve the mother’s health care-seeking behaviour.

## Background

Health care-seeking behaviour is an action taken by an individual to an internal and external stimulus to find a suitable solution after the child has a health problem.^1^ According to the World Health Organization (WHO), human health is complete physical, mental and social well-being not merely the absence of disease.^2,3,4^

Childhood illnesses are the main health problems globally, predominantly in Asian and African subcontinents.^5^ Around 6.3 million children aged under 5 years died in 2013 worldwide, which could be prevented with simple and inexpensive interventions.^6^ This is the most vulnerable group of the population suffering from common childhood illness.^6^ The global under-five mortality in 2013 was 41 per 1000 live births^6^ and 67 deaths per 1000 live births in Ethiopia in 2016.^7^

The children are at risk for malnutrition because of the problems in nutrition, socio-economic and health factors.^8^ Delay in proper care of mothers in modern health facilities is a major cause of child death all over the world especially in sub-Saharan Africa.^9,10^ Furthermore, poverty and lack of education are the major factors associated with under-five mortality in Ethiopia.^7^

According to the WHO, child mortality and morbidity could be reduced by 20% if there is appropriate health-seeking behaviour.^11^ Health-seeking behaviour is a function of not only the accessibility of health facilities and other sources of health care but also inspiration and capacity of individuals to seek medical treatment.^12^

Even though the common childhood illnesses are manageable successfully if timely recognised, facilitation of modern care-seeking behaviour remains a challenge today.^13^ A large number of children die without ever reaching a health facility.^10^ This is attributed to delays in seeking care by mothers.^10^ This delay affects child health and leads to child health complications that make medical care to be ineffective. Therefore, appropriate care-seeking behaviour is an important parenting instrument in preventing the child from preventable morbidity and moralities.^14^

To meet the Sustainable Development Goal (SDG) target *one* on under-five mortality by 2030 in reducing the number of under-5 deaths by 10 million between 2017 and 2030,^5^ rapid improvement is needed to raise the community awareness in modern health-seeking behaviour.

Although Ethiopia has universal access improvement on standard management of common childhood illnesses,^15^ modern care-seeking behaviour of caregivers for childhood illness remained low. In Shire town, the modern health-seeking behaviour and the associated factors remained unclear and insufficiently studied. Therefore, the aim of this study is to assess modern health-seeking behaviour and associated factors among mothers who have children under the age of five years in Shire town, Northwest Tigray, Ethiopia.

## Methods

### Study area

The study was conducted from March to April 2018. Shire town is located 1087 km from Addis Ababa, the national capital city of Ethiopia and 309 km from Mekelle, the regional capital city of Tigray. The town has two governmental hospitals, two governmental health care centres, and one private health care centre. According to the 2007 population census, the total population of Shire town was approximately 47 284.

### Study design

The community-based, cross-sectional study design was employed.

### Study populations

All mothers who have children under the age of five years in Shire town were the source population and who have children under the age of five years at randomly selected Kebeles (the smallest administrative unit in Ethiopia) was the study population and sampled mothers in the selected Kebeles (the smallest administrative unit in Ethiopia) were the study units. Mothers who are seriously ill, mentally ill and unable to speak and listen were excluded from the study. In this study, the dependent variable was modern health care-seeking behaviour for common childhood illness and the independent variables were sociodemographic factors, childhood factors, and service availability and accessibility.

### Sample size and sampling technique

The sample size was determined using single population proportion determination formula by taking the magnitude of health-seeking behaviour 72.7% from previous studies conducted in Bahir dar,^16^ 5% marginal error (d) with 95% confidence. The final sample size with the design effect of 1.5 and 10% non-response rate was 504. Multi-stage sampling technique was used for interviewing 504 study participants.

From the total five Kebeles, three Kebeles were selected by simple random sampling technique. To get the sample size from these three Kebeles (the smallest administrative unit in Ethiopia) proportional allocation to sample size was carried out. Then systematic sampling technique (*K* = 16) was used to choose the household for an interview. The first mother was selected using a lottery method. The household numbers were taken from health extension workers in Shire town a sampling frame was made from it.

### Data collection tools and procedure

Three nurses with a BSc degree collected data using a pre-tested interviewer-administered structured questionnaire. The tools were adapted from different works of literature^13,16,17,18^ then modified and rewritten to fit the local situation and research objective. To minimise errors in data entry double data entry was applied.

### Operational definition

#### Modern health care-seeking behaviour

Mothers/caregivers of sick children going to formal health care institutions (government health facilities and private hospitals/clinics) to seek care.^2^

#### Caregiver

The responsible individuals who take care of a child either the relatives of the child or the mother.^13^

### Data quality control

The training was given to data collectors regarding the objective of the study and data collection methods. The questionnaire was prepared in English then translated into Tigrigna (local language). The tool was pre-tested on 5% of the sample size in Aksum town. Continuous follow-up and supervision were made by the supervisor and principal investigator to check for completeness and consistency of the data.

### Data processing and analysis

The data were coded, entered, cleaned and edited using EPIDATA version 3.1, and then exported to Statistical Package for Social Science (SPSS) Version 22.0 for analysis. A binary logistic regression model was used to look at the statistical association between the outcome variable and every single independent variable. Variables that showed statistical significance during bivariate analysis at ≤ 20% (*p*-value ≤ 0.20) were entered into multivariate logistic regression by using the backward elimination method. The appropriateness of the model for analysis was checked by the Hosmer-Lemeshow test. Results were shown using tables and texts. The strength of associations was estimated by using adjusted odds ratios (AOR) with 95% CI and significance were declared at a *p*-value < 0.05.

### Ethical considerations

Ethical clearance was obtained from Aksum University, College of Health Science, Institutional Review Board (AKU-CHS, IRB) of the research committee. Official permission was also secured from the Tigray Regional Health Bureau. Then a permission and support letter was written to the health office of Aksum town. Respondents were informed about the purpose of the study, the information was collected after obtaining written consent from each participant. Written consent from all the informed respondents were needed before the start of each interview. Respondents were allowed to refuse or discontinue participation at any time they wanted. Information was recorded anonymously and confidentiality and beneficence were assured throughout the study.

## Results

### Sociodemographic characteristics of mothers or caregivers and children

About 504 mothers/caregivers were involved with the response completion rate of 100%. The majority of study participants, 492 (97.6%) were biological mothers of the child, 297 (59%) were equal to or over 28-years old and 294 (58.3%) were Orthodox Christians. In this study, 449 (88.9%) mothers/caregivers were married and 193 (38.3%) were housewives by occupation. Regarding the educational status, 167 (33.1) of the mothers and/or caregivers were in primary school (1st–8th Grade) and around 336 (66.7%) had less than or equal to five family members. Around 260 (51.6%) mothers/caregivers wanted a girl child. With regard to the children, 153 (30.4%) were in the age group of 0–11 months and the sex of the child was evenly distributed between female and male (Table 1).

**TABLE 1:** Sociodemographic characteristics of mothers/caregivers, and child in Shire Town, Tigray, Ethiopia, 2018.

Variables	Frequency	Percentage
**Primary caregiver of child**		
Mother	492	97.6
Out of mother	12	2.4
**Age**		
< 27 years	207	41.1
≥ 28 years	297	58.9
**Marital status**		
Married	449	89.1
Divorced	45	8.9
Widowed	10	2.0
**Occupation**		
Farmer	34	6.7
Governmental labour	147	29.2
Merchant	87	17.3
Daily labour	43	8.5
Housewife	193	38.3
**Religion**		
Orthodox	294	58.3
Protestant	75	14.9
Muslim	126	25.0
Other	9	1.8
**Educational status**		
Unable to read and write	60	11.9
Primary school	167	33.1
Secondary school	154	30.6
College and above	123	24.4
**Number of families**		
< 5	336	66.7
≥ 5	168	33.3
**Age of child**		
0–11 months	153	30.4
12–23 months	102	20.2
24–35 months	105	20.8
36–47 months	54	10.7
48–59 months	90	17.9
**Family preferred sex**		
Male	164	32.5
Female	142	28.2
Both	198	39.3
**Sex of child**		
Male	244	48.4
Female	260	51.6

### Health-seeking behaviour and health service utilisation of mothers in Shire town

In this study, 357 (70.8%) mothers were having greater than four antenatal care (ANC) follow-ups and 489 (97%) gave birth in health facilities. Around 335 (66.5%) children were exclusively breastfed for more than eight times (Table 2).

**TABLE 2:** Markers of modern health care-seeking behaviour associations in Shire town, North Ethiopia, 2018 (*n* = 504).

Variables	Frequency	Percentage
**ANC follow-up**		
< 4 times	147	29.2
≥ 4 times	357	70.8
**Maternal delivery**		
Health institution	489	97.0
Home	15	3.0
**Breastfeeding per day**		
< 8 times	169	33.5
≥ 8 times	256	50.8
**Duration of EBF**		
< 6 months	169	33.5
≥ 6 months	335	66.5

ANC, antenatal care; EBF, exclusive breastfeeding.

Overall, around 489 (97%) caregivers were facing one or more childhood illness and the common childhood illnesses complained by mothers were cough 261 (53.4%), fever 203 (41.5%) and diarrhoea 128 (26.2%). With respect to decision-making regarding the health of the child, mothers made 358 (71.0%) decisions. Overall, the magnitude of modern health care-seeking behaviour of mothers was 76.2% (72.1, 80). The main reason for those who did not seek medical care was stated as being the cost of medical care (41.2%) and perceived as perceived as a mild illness (29.4%). Around 103 (20.4%) of the caregivers delayed seeking modern health care for ≥ 3 days. The main reason for this was ‘thought the illness was mild/will resolve by itself’. Regarding the perception of illness, nearly half 236 (46.9%) of mothers perceived that the illness of their child was mild and around 283 (56.2%) mothers identified the severity of illness by viewing different symptoms (Table 3).

**TABLE 3:** Mothers’/caregivers’ modern health care-seeking behaviour from a child’s illness to initiate care in Shire Town, North Ethiopia, 2018 (*n* = 504).

Variables	Frequency	Percentage
**Illness of the child**		
Yes	489	97.0
No	15	3.0
**Reported symptom (more options) (*N* = 489)**		
Cough	261	53.4
Fever	203	41.5
Diarrhoea	128	26.2
Vomiting	94	19.2
Eye problem	57	11.7
Ear infection	39	7.8
Jaundice	23	4.7
Fast breathing	21	4.3
Itching	16	3.3
Cord swelling	10	2.0
**Was action taken to relieve the symptom? (*n* = 489)**		
Yes	472	96.5
No	17	3.5
**If yes what was the action (*n* = 472)**		
Health institution	384	76.2
Holy water	22	4.4
Traditional healer	4	0.79
Drug bought from a pharmacy	61	12.1
Both traditional and holy water	3	0.59
**Modern health-seeking behaviour**		
Yes	384	76.2
No	120	23.8
**If no, why not an action is taken**		
Waiting time	3	17.6
Cost of medical care	7	41.2
Illness was mild	5	29.4
Previous bad experience from professional	2	11.76
**Decision makers for medical treatment**		
Mother	358	71.0
Out of mother	146	29.0
**Perception of a mother about the severity of illness**		
Mild	236	46.9
Moderate	192	38.0
Severe	61	12.1
**Know severity**		
By any symptom	283	56.2
If the illness continuous long time	135	26.8
Loss of apatite	86	17.0
**The onset of seeking health care (*n* = 504)**		
One day	170	33.7
Two days	136	26.9
Three days	95	18.8
> 3 days	103	20.4
**Maternal perception on the cause of childhood illness (*n* = 504) (more option was possible)**		
Eating contaminated food and water	320	63.5
Microorganism	110	21.8
Lack of food	106	21.0
Teething	124	24.6
Evil eye	102	20.2
Curse of God	124	24.6

### Factors associated with modern health care-seeking behaviour of mothers in Shire town

In the binary logistic regression at a *p*-value of ≤ 0.2, caregiver’s age, caregiver level of education, family number, child feeding per day, decision-making, number of ANC follow-up, the perceived severity of illness and early detection of disease were statistically associated with modern health-seeking behaviour.

In multiple logistic regression by using the backward elimination method, caregivers with age of ≥ 28 years displayed more than 1.65 times health-seeking behaviour, than those with age of ≤ 27 years (AOR: 1.65; 95% CI [1.02, 2.68]). The odds that mothers with the educational level of secondary school and above were 0.44 times to have modern health-seeking behaviour than those with no education (AOR: 0.44; 95% CI [0.23, 0.86]). The caregivers who feed their child < 8 times per day seek 2.77 times more medical care than those who feed their child ≥ 8 times (AOR: 2.77; 95% CI [1.75, 4.38]). Caregivers who perceive their child’s illness as severe were two times higher to seek modern health care than mothers who perceive their child’s illness as mild (AOR: 2; 95% CI [1.07, 3.82]) (Table 4).

**TABLE 4:** Factors that affect health-seeking behaviour among mothers having under-five children in the Shire town, Northwest Ethiopia, 2018.

Variables	Health-seeking behaviour				Crude		Adjusted	
	Yes		No		OR	95% CI	OR	95% CI
	*N*	%	*N*	%				
**Age of the caregivers**								
≤ 27 years	168	81.2	39	18.8	1	-	1	-
≥ 28years	216	72.7	81	27.3	1.62	1.05, 2.49	1.7 5	1.07, 2.87*
**Level of education**								
No education	39	65	21	35	1	-	1	-
Primary school	122	73.1	45	26.9	2.22	1.21, 4.08	0.73	0.37, 1.44
Secondary and above	223	80.5	54	19.5	152	0.97, 2.4	0.44	0.23, 0.86*
**Family number**								
< 5	262	78.0	74	22.0	1.33	0.87, 2	1.06	0.62, 1.82
≥ 5	122	72.6	46	27.4	1	-	1	-
**Child feed per day**								
< 8 times	107	63.3	62	36.7	2.78	1.81, 4.22	2.77	1.75, 4.38
≥ 8 times	277	82.7	58	17.3	1	-	1	-
**Decision-making**								
Mother	279	77.9	79	22.1	1	-	1	-
Out of mother	105	71.9	41	28.1	1.38	0.89, 2.14	0.76	0.45, 1.27
**Number of ANC visit**								
< 4 times	103	70.1	44	29.9	1.58	1.02,2.44	1.35	0.82, 2.20
≥ 4 times	281	78.7	76	21.3	1	-	1	-
**Perceived severity of illness**								
Severe	37	60.7	24	39.3	2.54	1.39, 4.65	2	1.07, 3.82*
Moderate	159	82.8	33	17.2	0.81	0.49, 1.33	0.88	0.53, 1.48
Mild	188	79.7	48	20.3	1	-	1	-
**Early detection of disease is important**								
Yes	320	77.7	92	22.3	1.5	0.92, 2.51	0.72	0.4, 1.28
No	64	69.6	28	30.4	1	-	1	-

ANC, antenatal care; OR, Odds ratio; CI, confidence interval.

* , statically significant at *p*-value of < 0.05.

## Discussion

The aim of this study was to assess the modern health care-seeking behaviour of mothers and caregivers with children under the age of five years with childhood illness and to find out the associated factors. In this study, 76.2% of the caregivers sought modern health care when their child developed an illness. This finding is lower than the studies carried out in North West Ethiopia 84.4%,^19^ Dangila town 82.1%,^13^ Oromia region, Ethiopia 87%^20^ and urban slum 90%.^17^ The difference between these studies might be because of the difference in traditional practice and sociodemographics.

Modern health-seeking behaviour in this study is higher than the studies carried out in the rural Ensaro District, North Shoa Zone 59.9%^18^ and Yemen 51.4%.^10^ This difference could be because of the availability of health posts in all selected Kebeles of our study area. The urban population could utilise health service more than the rural population.^21^ In our study, all the participants were from an urban dwelling. This could be explained by the fact that the urban population could have access to information and, therefore, the caregivers could tend to seek care from nearby health facilities when the illnesses worsened. The other possible reason might be related to the expansion of urban health extension programmes that create awareness about early health-seeking behaviour.

The age of a caregiver is one predictor of modern health care-seeking behaviour. In our study, caregivers aged ≥28 years were 1.65 times more likely to have modern health-seeking behaviour than those aged ≤ 27 years. This is consistent the study carried out in Dangila town^13^ North West Ethiopia^19^ and in Nigeria.^22^ As the age of mother increases, their experience increases, which helps them to easily identify the common childhood illness and seek better medical care.

It is obvious that higher level of caregiver’s school education yields good modern health-seeking behaviour. School education could increase the mother’s knowledge about common health problems, and healthy habits. In contrast to this idea, in our study, the odds of modern health-seeking behaviour among caretakers in secondary school and above the educational level were 66% less likely to seek medical care than non-educated ones. This is inconsistent with the study carried out in Yemen.^10^ This might be because of the dwelling similarity among the caregivers. As all the respondents in our study were from the urban dwelling.

Our finding revealed that the caregivers who feed < 8 times a day were three times more likely to seek medical care than those who feed ≥ 8 times. This could make the mothers/caregivers aware that insufficient feeding could affect the child’s health; therefore, the mothers/caregivers could seek modern health care in health institutions timely.

In this study caregivers perceiving of illness as severe were strongly associated to seek medical care. In the current study, mothers who perceived illness as severe were two times more likely to seek medical care than those who perceive the illness as mild. This finding is similar to studies carried out in Dangilia,^13^ Sierra Leon,^23^ Nigeria,^24^ a systemic review performed in developing countries,^14^ in Yemen^10^ and Kenya.^14^ This could be because the more severe episodes of illness might increase the likelihood of visiting a health service. This might be because of the mother’s belief that the illness will improve by itself and she will wait until her child might show another symptom. Then this may put the children at risk of severe illness. This could be owing to the fact that 334 (66.3%) of mothers/caregivers bring their child after two days onset of the symptom.

## Conclusion

The overall modern health-seeking behaviour of mothers who have children under the age of five years were 76.2%. Age of the mother 28 years and above, who fed her child < 8 times per day and perceived the severity of illness were the significant factors for health-seeking. The health care provider should be aware that any illness in childhood could affect the child. Therefore, health care services should be strengthened at the community level by introducing all the new mothers to the urban health extension workers. These health workers should increase awareness among mothers/caregivers regarding the symptoms of illness and timely care-seeking.
